# Design of INSPIRE: Evaluation of the effectiveness of practice facilitation on implementation of screening, brief interventions, referral to treatment and medication for unhealthy alcohol use identification and treatment in primary care

**DOI:** 10.1016/j.conctc.2024.101413

**Published:** 2024-12-09

**Authors:** Jennifer Bannon, Justin D. Smith, Mark J. Van Ryzin, Megan McHugh, Jennifer Heinrich, Theresa L. Walunas, Abel N. Kho

**Affiliations:** a Department of Emergency Medicine, Feinberg School of Medicine, Northwestern University, Chicago, IL, USA; b Department of Population Health Sciences, Division of Health System Innovation and Research, Spencer Fox Eccles School of Medicine at the University of Utah, 295 Chipeta Way, Salt Lake City, UT, USA; c College of Education, Oregon Research Institute, University of Oregon, Eugene, OR, USA; d Division of General Internal Medicine and Center for Health Information Partnerships, Institute for Public Health and Medicine, Northwestern University Feinberg School of Medicine, USA; e Department of Medicine, Division of General Internal Medicine and Center for Health Information Partnerships, Institute for Public Health and Medicine, Northwestern University Feinberg School of Medicine, USA

**Keywords:** Brief intervention, Electronic health record, Implementation research, Practice-based research, Practice facilitation, Quality improvement, Unhealthy alcohol use

## Abstract

**Background:**

Unhealthy alcohol use is a leading cause of preventable mortality and a risk factor for an array of social and health problems. The Intervention in Small primary care Practices to Implement Reduction in unhealthy alcohol use (INSPIRE) study is part of a nationwide campaign to improve the identification and treatment of patients engaging in unhealthy alcohol use.

**Methods:**

We conducted a single arm, pragmatic study consisting of seventeen primary care practices in the Chicago metropolitan area, Wisconsin, and California across two waves with a 6-month latent period, a 12-month intervention period, followed by a 6-month sustainability period. Enrolled practices were independent, Federally Qualified Health Centers, network-based, and academic health centers. INSPIRE utilized the RE-AIM (Reach, Effectiveness, Adoption, Implementation, Maintenance) framework to examine implementation feasibility, fidelity, and performance of clinicians on practice adoption of screening, brief intervention, referral to treatment (SBIRT) and medication for unhealthy alcohol use (MAUD) activities in primary care clinics.

**Results:**

Seventeen eligible primary care practices were enrolled over the course of 21 months beginning in March of 2020 through December of 2021. There was a pause in recruitment from March of 2020 through July of 2020 due to the Covid-19 pandemic. The majority of enrolled practices were small (<6 clinicians) and were part of a network. 57 % of clinicians completed the educational modules in part or in full.

This paper will outline the INSPIRE protocol and design. Additionally, we will present practice demographic data, recruitment data and results related to on-line learning module completion.

**Conclusion:**

The INSPIRE study will evaluate the ability of primary care clinicians in small practices to participate in practice education programs and implement standard screening and treatment protocols, adapted for documentation in the electronic health record (EHR). The study will also identify the factors that facilitated or hindered improvement and sustainability using quantitative and qualitative analysis methods.

## 1 Background

Unhealthy alcohol use is a leading cause of preventable mortality and a risk factor for an array of social and health problems with an estimated annual economic impact of $249 billion. Despite the significant public health impact of unhealthy alcohol use, and endorsement by the US Preventive Services Task Force, rates of screening across the country remain low [1]. In Illinois and Wisconsin, two states with the highest levels of unhealthy alcohol use in the Midwest, less than 17 % of patients receive screening in a primary care setting and only 5 % of patients with reported heavy alcohol use receive treatment [2].

Effective screening and treatment in small practices is limited due to a lack of clinician education around screening and management, low rates of prescribing medications for alcohol use disorder, poor availability of referral programs, and difficulty integrating interventions into existing clinical workflows [3]. Checklist-based screening tools present a simple, easy to understand strategy and have been successfully applied within the healthcare setting [4].

The USPSTF reports evidence that screening patients in primary care settings can be effective to identify patients with unhealthy alcohol risk factors. The Alcohol Use Disorders Identification Test (AUDIT) is an evidence-based screening tool for detecting unhealthy alcohol risk [1]. Additionally, brief behavioral counseling interventions with follow-up care produce small to moderate reductions in alcohol consumption that are sustained over 6–12 months or longer [4].

Brief intervention counseling delivered by primary care providers, therapists, and research staff can decrease alcohol use for at least 1 year in nondependent drinkers in primary care clinics, managed care settings, hospitals, and research settings [3].

Referral to treatment is recommended when patients meet the diagnostic criteria for substance dependence or other mental illnesses as defined by the Diagnostic and Statistical Manual of Mental Disorders, Fourth Edition (DSM-IV) [5]. Research findings suggest that motivational-based BIs can increase patient participation and retention in substance abuse treatment [6].

An 10.13039/100000133Agency for Healthcare Research and Quality (10.13039/100000133AHRQ) review that included 135 studies of pharmacologic treatment of alcohol use disorders (AUD) in outpatient settings found moderate evidence to support the use of naltrexone and acamprosate [5]. Acamprosate and naltrexone reduce alcohol consumption and increase abstinence rates, although the effects are modest [5].

Pharmacotherapies can complement psychosocial treatment by countering one or more of the neurobehavioral mechanisms that initiate and maintain alcohol use. For example, medications such as disulfiram and opioid antagonists counter the positively reinforcing stimulant effects of alcohol and increase its aversive effects [5].

In the INSPIRE project, we aim to test the feasibility and effectiveness of office-based screening supported by behavioral (screening, brief intervention, referral to treatment (SBIRT)) and medication-based interventions (medication for unhealthy alcohol use (MAUD)) in primary care practices, through an integrated platform of education, practice facilitation, and embedded electronic health record (10.13039/100000081EHR) technology. The project brings together a complementary team of experts from academic institutions, a quality improvement (QI) organization, practice-based research networks and independent research organizations, a majority of whom have previously worked together to implement similar office-based interventions supported by practice facilitation to improve cardiovascular care [7].

The INSPIRE study protocol has the following aims:

**Aim 1:** Develop and implement online continuing medical education (CME) programs to support the in-clinic implementation of behavioral intervention and medication for alcohol use disorder for people with unhealthy alcohol use and identify patients who may benefit from further referral.

Research question: What was the reach of the CME online modules to primary care clinicians in enrolled practices?

**Aim 2:** Conduct a two-wave study in small primary care practices to determine whether practice facilitation can affect health care provider behavior change and increase the adoption and use of an EHR-based checklist to improve screening and treatment for people with unhealthy alcohol use.

Research question: What is the impact of practice facilitation on clinician behavior change and adoption and use of SBIRT screening and treatment for people with unhealthy alcohol use.

**Aim 3:** Evaluate the ability of health care providers in small practices to participate in practice education programs and implement and sustain standard screening and treatment protocols adapted for documentation in the EHR and identify the factors that facilitated or hindered improvement and sustainability using quantitative and qualitative analysis methods.

Research question: What type of evaluation tools can assist with the measurement of practice and provider adoption and implementation of the INSPIRE strategies?

This design paper focuses on the goals stated above, the introduction of the INSPIRE implementation toolkit and interventions (SBIRT and MAUD), and describes the recruitment strategy, practice facilitation strategy and describes the design to accomplish the aims.

## 2 Methods

### 2.1 Study setting and participants

The INSPIRE team is a collaborative composed of eight organizations based in Illinois and Wisconsin with experience in practice engagement, effective use of EHR technology, QI research, practice transformation, and substance use screening and management.

INSPIRE builds on an infrastructure developed through the Agency for Healthcare Research and Quality (AHRQ)-funded Healthy Hearts in the Heartland study that was part of the EvidenceNOW (program to improve evidence-based cardiovascular care in primary care practices, leveraging practice facilitation and EHR technology).

INSPIRE is led by Northwestern University, and includes partners MetaStar, Inc. (the Wisconsin Health IT Regional Extension Center and Quality Improvement Organization), Alliance Chicago (a health center controlled network and practice-based research network), ACCESS Community Health Network (a practice-based research network with experience in substance use management) and Northwestern Medicine (a health system-based practice network) and will expand the collaboration to include and three research institutes with experience in substance use education and program evaluation: the American Institutes for Research, the Oregon Research Institute, and the Altarum Institute.

**Fig. 1:**
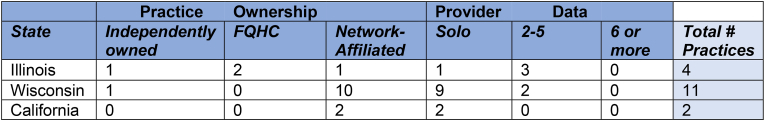
Enrolled practice demographics.

The INSPIRE interventions were delivered by practice facilitators and measured at the practice level. The consented participants were clinic providers. Providers include physicians, physician assistants and advanced nurse practitioners. All practice staff participated in the INSPIRE SBIRT/MAUD interventions. We recruited twenty-three eligible primary care practices over the course of 21 months beginning in March of 2020 through December of 2021(Fig. 1).

### 2.2 Study design

The INSPIRE team developed and deployed a robust online and in-person learning collaborative to support the in-clinic implementation of SBIRT and MAUD for patients with unhealthy alcohol use and those that may benefit from further referral for unhealthy alcohol use.

We conducted and assessed the implementation of a single-arm, pragmatic study with a staggered rollout design in small primary care practices to determine the adoption and efficacy of an EHR-based workflow to improve screening and treatment for people with unhealthy alcohol use. The design was adjusted to accommodate recruitment and intervention delivery interruptions caused by the COVID-19 pandemic. The adjustment resulted in Wave 2 practices having a four-month pre-intervention phase rather than the planned six-month pre-intervention phase (Fig. 5). No power analysis was conducted.

We will evaluate the ability of small practices in our region to participate in practice education programs, implement and sustain standard screening and treatment protocols adapted for documentation in the EHR and identify the factors that facilitated or hindered improvement and sustainability using quantitative and qualitative analysis methods.

### 2.3 Procedures

#### 2.3.1 Primary care practice recruitment

The INSPIRE study-initiated practice recruitment in March of 2020. Recruitment was halted for five months due to infection mitigation-related disruptions to primary care as a result of the COVID-19 pandemic. Recruitment resumed in August of 2020. The INSPIRE study's practice recruitment strategy and tools (including fliers, social media announcements and web pages) were developed and updated to reflect COVID-19 pandemic concerns (e.g., virtual practice facilitation options) and the data collection system for tracking recruitment was documented in the customer relationship management (CRM) system Facilitation Activity and Intervention Tracking System (FACITS).

Recruitment methods included minimal in-person recruitment, the reengagement of providers and practices from prior research and QI projects, as well as emails, newsletters, and faxes. Due to the COVID-19 pandemic our research team was unable to meet recruitment goals established in our protocol. Our recruitment strategy also included newsletters and outreach that aligned the pandemic with documented increases in alcohol use.

**Fig. 2:**
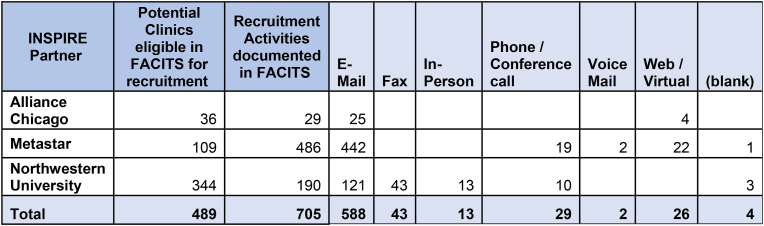
Recruitment.

We recruited 23 practices, 17 of which remained engaged through the intervention and implementation phase of the study (Fig. 2).

#### 2.3.2 Implementation strategies

Several implementation strategies—actions taken to improve utilization of an intervention at the health system or provider level [8] were used to support clinicians and primary care practices in adopting and implementing the unhealthy alcohol use screeners and interventions.

##### 2.3.2.1 Clinician education strategy

**Fig. 3:**
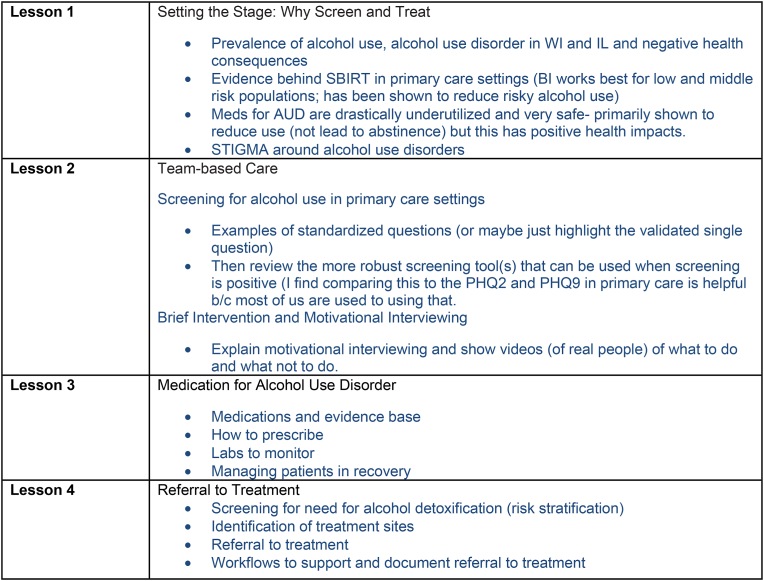
Content of CME learning modules.

**Fig. 4:**
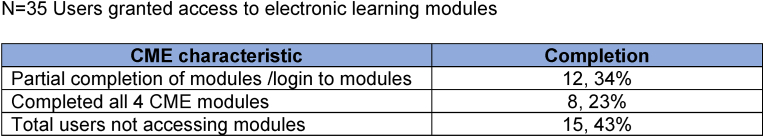
REACH of CME learning modules N = 35 Users granted access to electronic learning modules.

Our INSPIRE team, consisting of a clinical champion and INSPIRE practice facilitators, developed four online CME education programs to support the in-clinic implementation of behavioral intervention and medication for alcohol use disorder for people with unhealthy alcohol use and identify patients who may benefit from further referral. The four learning modules covered the following sections: Screening, Brief Intervention, Medications for Unhealthy Alcohol Use and Referring to external counseling (Fig. 3). Clinicians and practice staff were offered online access to all modules (Fig. 4). Continuing education credits were offered upon completion of each separate module for clinicians. The practice facilitation team was also provided access to the modules to strengthen SBIRT knowledge and align the intervention with the module content.

**Fig. 5:**
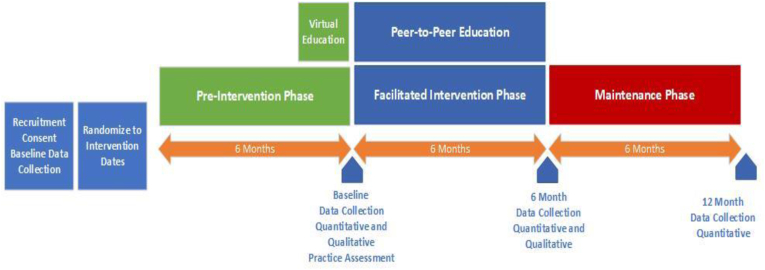
Study Design schedule for individual practices in INSPIRE study.

##### 2.3.2.2 Practice facilitation

Practice facilitation was provided by a total of five practice facilitators, including a practice facilitation lead. The practice facilitation team was part of a larger national collaborative community that met monthly to share tools, best practices and receive education on desired SBIRT/MAUD initiatives. Practice facilitators also utilized a toolkit developed by the lead and the national collaborative to support unhealthy alcohol use QI initiatives and practice-tailored implementation strategies.

Practice facilitation activities consisted of in-person facilitation, remote facilitation, and a hybrid strategy whereas both methods were utilized by the practice facilitators in the deployment of INSPIRE interventions. Implementation strategies included the following: QI training and support, 10.13039/100000081EHR optimization, practice performance monitoring guidance, team-based care strategies, and motivational interviewing.

Practice facilitation components included:

• Assessing and documenting relevant electronic data for unhealthy alcohol use process measures
• Documentation of unhealthy alcohol use utilizing validated Alcohol Use Disorders Identification Test (AUDIT [3] screening tools in appropriate standardized EHR fields to support valid data extraction
• Modify documentation processes to ensure SBIRT data is recorded for extraction
• Validate data extracts to ensure appropriate capture of patient and provider quality data
• Establish ongoing data validation and review processes

##### 2.3.2.3 Other implementation strategies

In addition to the CME modules and practice facilitation, the INSPIRE implementation toolkit also consisted of 1) posters to “normalize” screening for alcohol use in practice; 2) patient education tools; and 3) a provider handbook to improve sustainability of the interventions throughout our region to support reduction of unhealthy alcohol use.

### 2.4 Data collection

Data collection from the consented practices for INSPIRE was captured via the REDCap platform [9], in (FACITS) [10], and in Quickbase at baseline, 6 months(post-intervention) and at 12 months to measure sustainability. Surveys were developed by the research team and administered at the practice level to collect data including practice demographics, change capacity, SBIRT interventions, and practice climate. Surveys were collected in REDCap and practice facilitation activities were logged into FACITS.

Practice facilitation activity components included:

• Assessing and documenting relevant electronic data for unhealthy alcohol use process measures
• Documentation of unhealthy alcohol use utilizing validated AUDIT screening tools in appropriate standardized fields to support valid data extraction
• Modify documentation processes to ensure SBIRT data was recorded for extraction
• Validate data extracts to ensure appropriate capture of patient and provider quality data
• Establish ongoing data validation and review processes

One study outcome was to determine the number of QI interventions each practice planned to implement; this may vary depending upon prior QI strategies in place, practice capacity and engagement throughout the pandemic. We also determined the number of interventions actually attempted. For each strategy attempted, practice facilitators rated the degree of implementation at the end of the 6-month implementation period in Quickbase. Barriers to implementation were also documented by the practice facilitation team in the (CRM) system. Tracking data was documented by practice facilitators in the CRM at the time of each visit.

### 2.5 Study measures

#### 2.5.1 Data collection concerning primary care practices and clinicians

INSPIRE evaluated the ability of clinicians in primary care practices in our designated regions to participate in practice-based education to promote the implementation and sustainment of standard screening and treatment protocols adapted for documentation in the EHR. INSPIRE also identified the factors that facilitated or hindered improvement and sustainability using quantitative and qualitative analysis methods.

Assessment of continuing medical education (CME) credits earned and completion of each module were measured to determine the effectiveness of electronic learning education as an intervention in the INSPIRE study.

Reach of the CME modules (Aim 1) was measured by: Number (and proportion) of providers who accessed the CME modules and number (and proportion) of providers who completed the CME modules. Implementation and uptake of the online modules was also tracked as described in this section.

We assessed the amount of time facilitators spent with practices, what SRIRT/MAUD facilitation activities were performed by the practice staff and the practice facilitator's assessment of practice engagement in the interventions.

##### 2.5.1.1 Practice demographic survey

The 16-item practice survey is completed at baseline. This survey is completed by a practice champion and other practice staff that have knowledge of practice and patient demographics. This data was inputted by a designee from the practice (not necessarily a provider). This survey was distributed via email link and is completed directly into Research Electronic Data Capture (REDCap) [9] Intervention tracking elements were assessed at baseline, 6 months, and 12 months. The demographic data is generated from EHR reporting. Variables include patient demographics, staff demographics, payer mix, clinic type (i.e. network-affiliated, federally qualified health system, provider-owned), and full-time equivalent (FTE) status.

##### 2.5.1.2 SBIRT/MAUD survey

This 13-item survey [3] collects data related to interventions that are in place related to alcohol use screening, brief intervention, referral to treatment, and medications for alcohol use disorder. This assessment will help with identifying interventions based on current workflow. The survey is completed by a practice champion or other practice staff that understand the current workflows of SBIRT and MAUD. This survey was administered by the research team via email at baseline, 6 months, and 12 months.

##### 2.5.1.3 Change Process Capability Questionnaire (CPCQ)

The goal of the 14-item CPCQ (cite) is to assess the change capacity of a practice. These questions were completed by one senior member of the practice who has good insights into the clinical operations of the practice, such as a lead clinician or an office manager. Change processes include covert and overt activities and experiences that individuals engage in when they attempt to modify problem behaviors. Many studies have shown that successful self-changes use different processes during distinct stages of change [11]. Completion of this survey is essential to help the INSPIRE evaluation team understand barriers to the unhealthy alcohol use intervention and barriers in the practice culture. Capacity for QI was measured at baseline, after a 6-month implementation period, and 12 months after randomization (the sustainability period).

##### 2.5.1.4 Implementation climate scale (ICS)

The ICS [8] assesses the degree to which there is a strategic organizational climate supportive of evidence-based practice implementation. Implementation climate is defined as employees’ shared perceptions of the policies, practices, procedures, and behaviors that are rewarded, supported, and expected in order to facilitate effective evidence-based practice (EBP) implementation.

##### 2.5.1.5 Implementation leadership scale (ILS)

The 10.13039/100023424ILS [8] is a brief and efficient measure that can be used for research or organizational development purposes to assess leader behaviors and actions that actively support effective EBP implementation.

##### 2.5.1.6 Intervention tracking (FACITS)

**Fig. 6:**
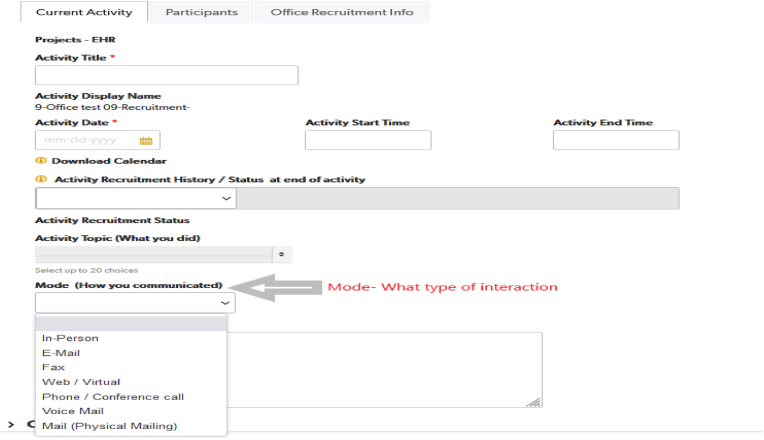
Practice Facilitation documentation and Intervention Tracking Example in FACITS-Quickbase CRM.

The practice facilitators recorded recruitment and practice facilitation activities in the INSPIRE FACITS module in the Quickbase CRM. PFs were responsible for documenting all interactions including type of encounter (e.g., virtual, in-person, phone, email, encounter start and end time (dosage), SBIRT interventions (Intervention tracking), and facilitation activities. Additionally, barriers to intervention completion and process measures were also captured in FACITS (Fig. 6).

#### 2.5.2 Data collection concerning patients

Study measures/metrics were selected by the national AHRQ collaborative team to standardize measurement outcomes among the unhealthy alcohol use study participants. All INSPIRE practices were not able to generate electronic clinical quality measure measurements to extract for outcome measurement. Practice facilitation activities in INSPIRE practices included EHR optimization and data standardization to assist with SBIRT data collection. Number of patients aged ≥18 years with a patient encounter during the past 3 months. Exclude any patients who have a current diagnosis of or who are seeking treatment for alcohol abuse or dependence.

1) The number and percent of patients aged ≥18 years with a patient encounter during the past 3 months AND who were screened for unhealthy alcohol use using a systematic screening method at least once within the past 3 months.
2) Number and percent of patients aged ≥18 years with a patient encounter during the past 3 months who were screened for unhealthy alcohol use using a systematic screening method at least once within the past 3 months AND who screened positive for unhealthy alcohol use.
3) Number and percent of patients aged ≥18 years with a patient encounter during the past 3 months who screened positive for unhealthy alcohol use using a systematic screening method AND who received brief counseling.
4) Number and percent of patients aged ≥18 years with a patient encounter during the past 3 months who have a likely alcohol use disorder AND who initiated medication-assisted therapy.
5) Number and percent of patients aged ≥18 years with a patient encounter during the past 3 months who screened positive for unhealthy alcohol use using a systematic screening method and were referred to specialty care for alcohol treatment. This metric was optional for grantees to report.

### 2.6 Statistical analysis plan/process measures

We planned to calculate the percentage of eligible patients in a practice who met criteria for improved screening and treatment outcomes. This is calculated as the number of patients who were screened for unhealthy alcohol at least once within the past 3 months, divided by the number of patients aged 18 or more years with a patient encounter during the past 3 months (excluding any patients who have a current diagnosis of or who are seeking treatment for alcohol abuse or dependence). In our sample, there were six practices that submitted performance data across Time 0-Time 2; four other practices provided data but only at T0 and thus were excluded from further analysis. These 6 practices varied substantially in their screening frequency at baseline (M = .17, SD = .24, Range = .00 to .54) and mean percentages increased at T1 (M = .30, SD = .27, Range = .02 to .67) and T2 (M = .58, SD = .34, Range = .12 to 1.00). We planned to conduct a growth analysis using RM-ANOVA to evaluate whether there was significant change over time.

We also planned to calculate the capacity for quality improvement (QI) across our practices. This was measured at baseline, after 6-month implementation period, and 12 months after randomization (the sustainability period) using the Change Process Capability Questionnaire (Solberg et al., 2008). Scores were calculated as directed (N = 14 items, Cronbach's alpha = .96). Ten practices responded to the survey across all three time points, and these 10 practices varied substantially in their responses at baseline (M = .77, SD = .69, Range = .07 to 2.00) and declined at T1 (M = .54, SD = 1.08, Range = −1.29 to 1.71) and T2 (M = .38, SD = .78, Range = −.50 to 1.50). Of the remaining 7 practices, 3 provided data at T0 and T1, two provided data at T0 and T2, one provided data at T0 only, and one provided data at T2 only. As with the screening data, we planned to conduct a growth analysis using RM-ANOVA to evaluate whether there was significant change over time.

Finally, we planned to extend our RM-ANOVA for screening data to evaluate whether baseline CPCQ scores were a significant predictor of change over time (i.e., RM-ANCOVA).

## 3 Discussion

In this paper, we explain the goals and design for the INSPIRE study. At the end of the recruitment phase, 489 practices were contacted for participation through a variety of methods (see Fig. 3). 467 declined to participate in the INSPIRE study for varied reasons. Twenty-two practices initially consented to participate, however over the course of six months, five more practices withdrew before the start of the intervention phase. Seventeen practices proceeded to engage in the INSPIRE study.

A qualitative study based on a 5-question questionnaire was conducted prior to the intervention phase to understand recruitment barriers for quality improvement. 109 respondents indicated staff turnover, staffing shortages, and general time constraints, exacerbated by the pandemic prevented participation [12].

Most enrolled practices were solo providers, and most clinics associated with being part of a network system.

## 4 Conclusion

In this manuscript we describe the design, study goals and aims for the INSPIRE study, a three-year research program to help primary care practices implement and maintain evidence-based unhealthy alcohol use interventions. The COVID-19 pandemic impacted our recruitment goals and intervention uptake. Our study design was modified by the research team to accommodate a shorter pre-intervention phase for the second Wave in the study.

The recruitment and practice facilitation barriers that occurred during the COVID-19 pandemic will assist us in future research studies to adjust our study design to accommodate unforeseen changes in healthcare settings and cultures. Our study team successfully implemented SBIRT interventions in seventeen practices. Our work may be translatable to other clinicians and practice staff planning to adopt evidence-based substance abuse interventions in practice and to other research teams who may require rapid modifications to a study deign due to unforeseen circumstances in the healthcare landscape. Although our study was underpowered, we are confident that our pre-intervention data collection, study interventions and design modifications will support our aims.

## CRediT authorship contribution statement

**Jennifer Bannon:** Writing – review & editing, Writing – original draft, Resources, Formal analysis. **Justin D. Smith:** Writing – review & editing, Writing – original draft, Investigation, Formal analysis, Data curation. **Mark J. Van Ryzin:** Writing – review & editing, Supervision, Formal analysis, Data curation, Conceptualization. **Megan McHugh:** Writing – review & editing, Writing – original draft, Investigation, Formal analysis. **Jennifer Heinrich:** Resources, Project administration. **Theresa L. Walunas:** Writing – review & editing, Writing – original draft, Supervision, Resources, Methodology, Investigation, Funding acquisition, Formal analysis, Data curation, Conceptualization. **Abel N. Kho:** Writing – review & editing, Writing – original draft, Supervision, Resources, Methodology, Investigation, Funding acquisition, Formal analysis, Data curation, Conceptualization.

## Declarations

The authors declare that they have no known competing financial interests or personal relationships that could have appeared to influence the work reported in this paper.

## Funding

Funded by the 10.13039/100000133Agency for Healthcare Research and Quality Grant Number: 5R18HS027088-03.

## Declaration of competing interest

The authors declare that they have no known competing financial interests or personal relationships that could have appeared to influence the work reported in this paper.
