# Supplementary material for: Design of INSPIRE: Evaluation of the effectiveness of practice facilitation on implementation of screening, brief interventions, referral to treatment and medication for unhealthy alcohol use identification and treatment in primary care
Source: Contemp Clin Trials Commun. 2024 Dec 9;43:101413. doi: 10.1016/j.conctc.2024.101413 (PMC11718291; doi:10.1016/j.conctc.2024.101413)
Supplement: Multimedia component 1 [file mmc1.pdf]

Practice Level Survey Basic Characteristics Of Part

Please complete the survey below.

Thank you!

Instructions: Please complete the following 16-item survey, which is designed to collect information about your practice for the AHRQ Unhealthy Alcohol MW-Midwest study. In answering these questions, please refer to the status at the start of the quality improvement effort. We suggest you designate an Office Manager, Lead Clinician, or Practice Facilitator/Coach to complete this survey. We strongly encourage you to consult with others in your practice (e.g., Medical Director, Billing Manager) to obtain accurate information to complete this survey.

Today's date ________________________________

1. Grantee MW: Illinois/Wisconsin

2. Practice Site ID ________________________________

3. Indicate zip-code of practice ________________________________

4. Please indicate all members of the practice that you consulted with to complete this survey. (Check all that apply)

- ☐ Clinician (MD, DO, NP, PA)
- ☐ Behavioral health clinician (e.g., psychologist, counselor, social worker, licensed clinical social worker, licensed mental health counselor, chemical dependence and addiction counselor)
- ☐ Other clinical staff providing direct patient care (e.g., RN, LPN/LVN, medical assistant, certified medical assistant, medical technician)
- ☐ Office manager
- ☐ Front/back-office staff (those in practice operations and not directly involved in patient care, e.g., receptionists, appointment schedulers, billing staff, data analysts, etc.)
- ☐ Peer provider (e.g., certified peer specialist, peer support specialist, peer recovery coaches)
- ☐ Pharmacist (e.g., PharmD, clinical pharmacist, pharmacy technician)
- ☐ Other ______

5. Which of the following best describes your practice ownership? (Check all that apply)

- ☐ Clinician-owned solo or group practice
- ☐ Hospital/Health system owned
- ☐ Health maintenance organization (e.g., Kaiser Permanente)
- ☐ Federally Qualified Health Center or Look-Alike
- ☐ Non-federal government clinic (e.g., state, county, city, public health clinic, etc.)
- ☐ Academic health center/faculty practice
- ☐ Federal (Military, Veterans Administration, Department of Defense)
- ☐ Rural Health Clinic
- ☐ Indian Health Service
- ☐ Other ______

6. Please provide the total number of individuals, and combined Full-Time Equivalent (FTE) for each of the following staff members in your primary care practice. For individuals who fall in more than one category, please select the most appropriate role. If none, enter 0.

Staff Member Total Number Combined FTE
Primary care clinicians (MD, DO, NP, PA; excluding psychiatrists) ______ ______
Psychiatrists ______ ______
Behavioral health clinicians (e.g., psychologists, counselors, social workers, licensed clinical social workers, licensed mental health counselors, chemical dependence and addiction counselors) ______ ______

7. Please provide the total number of individuals for each of the following staff members in your primary care practice. For individuals who fall in more than one category, please select the most appropriate role. If none, enter '0.'

Staff Member Total Number
Other staff providing direct patient care (e.g. RN, LPN/LVN, medical assistant, certified medical assistant, medical technician) ______
Front/back-office staff (those in practice operations and not directly involved in patient care, e.g., receptionists, appointment schedulers, billing staff, data analysts, etc.) ______
Peer providers (e.g. certified peer specialists, peer support specialists, peer recovery coaches, etc.) ______
Pharmacists (PharmD, clinical pharmacists, pharmacy technicians) ______
If other, please specify ______ ______

8. Do clinicians from your practice provide inpatient care when patients from the practice are admitted to the hospital?

◯ Yes. Clinicians provide inpatient care
◯ No. Clinicians visit patients in hospital but do not provide inpatient care
◯ No. Hospital-based staff provides all care for inpatients

9. Is your practice recognized or accredited as a patient-centered medical home (PCMH)?

◯ Yes ◯ No

10. On average, how many patients does a full-time clinician in your practice see on a typical day?

____

11. Does your practice collect information on patient race?

◯ Yes ◯ No

Please give the percentage of your patients in the following categories, if your practice collects this information (should add to 100%). If N/A, please enter 0 in your responses.

White

______ %

Black/African American

______ %

American Indian or Alaska Native

______ %

Asian

______ %

Native Hawaiian or Other Pacific Islander

______ %

Other Race/Mixed Race

______ %

Percent Unknown

______ %

Total ______ %
Remaining ______ %

Warning: total doesn't equal 100%. If N/A, please enter 0 in your responses.

Please try again.

---

12. Does your practice collect information on patient ethnicity (e.g., Hispanic or Latino/Latina/Latinx)?

◯ Yes ◯ No

Please give the percentage of your patients in the following categories, if your practice collects this information: (should add to 100%). If N/A, please enter 0 in your responses.

Hispanic or Latinx ______ %
Non-Hispanic or non-Latino ______ %
% unknown ______ %

Total ______ %
Remaining ______ %

Warning: total doesn't equal 100%. If N/A, please enter 0 in your responses.

Please try again.

---

13. Please give the percentage of your patients in the following age categories: (should add to 100%). If N/A, please enter 0 in your responses.

0-17

______ %

18-39

______ %

40-59

______ %

60-75

______ %

76 and over

______ %

Total

______ %

Remaining

______ %

Warning: total doesn't equal 100%. If N/A, please enter 0 in your responses.

Please try again.

14. Does your practice collect the percentage of your patient's Gender Identity (e.g., men, women)?

◯ Yes ◯ No

Please give the percentage of your patients who identify as the following, if your practice collects this information (should add to 100%). If N/A, please enter 0 in your responses.

Men (including transgender men)

______ %

Female (including transgender women)

______ %

Non-binary, gender nonconforming, third gender, gender-fluid

______ %

Prefer not to self-describe or unknown

______ %

Total ______ %
Remaining ______ %

Warning: total doesn't equal 100%. If N/A, please enter 0 in your responses.

Please try again.

15. Please give the approximate percentage of your patients in the following payer categories: (should add to 100%). If N/A, please enter 0 in your responses.

Medicare only

______ %

Medicaid only

______ %

Dual Medicare and Medicaid

______%

Private or commercial

______ %

No insurance

______ %

Other

______ %

Total

______ %

Remaining

______ %

Warning: total doesn't equal 100%. If N/A, please enter 0 in your responses.

Please try again.

---

16. How did you obtain answers to questions 11-15?

☐ From EHR query/extract
☐ Through manual count
☐ Best estimate/informed approximation

Thank you for completing the INSPIRE Practice-Level Survey!

Change Process Capability Questionnaire

Today's date

Practice ID

Please indicate the extent to which you agree or disagree that your practice has used the following strategies to improve Unhealthy Alcohol Use care.

	Strongly disagree	Somewhat disagree	Neither agree or disagree	Somewhat agree	Strongly agree
1. Providing information and skills-training	○	○	○	○	○
2. Use of opinion leaders, role modeling, or other vehicles to encourage support for changes	○	○	○	○	○
3. Changing or creating systems in the medical group/clinic that make it easier to provide high quality care	○	○	○	○	○
4. Removal or reduction of barriers to better quality of care	○	○	○	○	○
5. Organizing people into teams focused on accomplishing the change process for improved care	○	○	○	○	○

	Strongly disagree	Somewhat disagree	Neither agree or disagree	Somewhat agree	Strongly agree
6. Delegating to non-physician staff the responsibility to carry out aspects of care that are normally the responsibility of physicians	○	○	○	○	○
7. Providing to those who are charged with implementing improved care the power to authorize and make the desired change	○	○	○	○	○
8. Using periodic measurement of care quality for the purpose of assessing compliance with any new approach to care	○	○	○	○	○
9. Reporting measurements of practice performance for comparison with their peers	○	○	○	○	○
10. Setting goals and benchmarking rates of performance quality at least yearly	○	○	○	○	○

	Strongly disagree	Somewhat disagree	Neither agree or disagree	Somewhat agree	Strongly agree
11. Customizing the implementation of any care changes to each site of care	○	○	○	○	○
12. Use of rapid cycling, piloting, pre-testing, or other vehicles for reducing the risk of negative results from introducing organization-wide change in care	○	○	○	○	○
13. Deliberately designing care improvements so as to make physician participation less work than before	○	○	○	○	○
14. Deliberately designing care improvements to make the care process more beneficial to the patient	○	○	○	○	○

Thank you for completing the INSPIRE Change Process Capability Questionnaire (CPCQ) survey!

Implementation Climate Scale (ICS)

Please complete the following 9-item measure, which assesses the degree to which there is a strategic organizational climate supportive of evidence-based practice implementation.

Today's date ______________________

Practice ID ______________________

Please indicate the extent to which you agree with each statement

	Not at all	Slight extent	Moderate extent	Great extent	Very great extent
1. This practice/clinic provides conferences, workshops, or seminars focusing on evidence-based practices	◯	◯	◯	◯	◯
2. This practice/clinic provides evidence-based practice trainings or in-services	◯	◯	◯	◯	◯
3. This practice/clinic provides evidence-based practice training materials, journals, etc	◯	◯	◯	◯	◯

	Not at all	Slight extent	Moderate extent	Great extent	Very great extent
4. Clinicians in this practice/clinic who use evidence-based practices are seen as clinical experts	◯	◯	◯	◯	◯
5. Clinicians who use evidence-based practices are held in high esteem in this practice/clinic	◯	◯	◯	◯	◯
6. Clinicians in this practice/clinic who use evidence-based practices are more likely to be promoted	◯	◯	◯	◯	◯

	Not at all	Slight extent	Moderate extent	Great extent	Very great extent
7. This practice/clinic provides financial incentives for the use of evidence-based practices	◯	◯	◯	◯	◯

8. The better you are at using evidence-based practices, the more likely you are to get a bonus or a raise	◯	◯	◯	◯	◯
9. This practice/clinic provides the ability to accumulate compensated time for the use of evidence-based practices	◯	◯	◯	◯	◯

Thank you for completing the INSPIRE Implementation Climate Scale (ICS) survey!

Implementation Leadership Scale (ILS)

Please complete the following questions.

Today's date __________________________________

Practice ID __________________________________

Do you serve in a leadership role in this facility (for example, acting as a mentor or in a supervisory role)?

◯ Yes
◯ No

If so, what role? __________________________________

Has the leadership in this facility changed within the last 6 months? ◯ Yes ◯ No

Has the leadership changed within the last 12 months? ◯ Yes ◯ No

The following 6 item survey assesses support in your practice for implementing evidence-based alcohol use interventions. Please complete the 6 items.

SBIRT and MAT Implementation Checklist

Please complete the following survey, which is designed to collect information about your practice for the Unhealthy Alcohol Use initiative. This survey is intended to be administered once at the start of the initiative, at 6 months, and at the end of the study. We strongly encourage you to consult with others in the practice (e.g., physicians, nurses, other staff) to obtain accurate information to complete this survey.

The SBI/RT and MAT Implementation Checklist is an assessment to see what interventions are in place related to alcohol use screening, brief intervention, referral to treatment, and medications for alcohol use disorder. This assessment will help with identifying interventions based on the current workflow.

Today's Date ________________________________

1. Grantee - Midwest

2. Practice ID ________________________________

Please indicate the extent to which you agree or disagree with each statement.

	Not at all	Slight extent	Moderate extent	Great extent	Very great extent
1. I support clinicians' efforts to learn about evidence-based alcohol use interventions	○	○	○	○	○
2. I support clinicians' efforts to use evidence-based alcohol use interventions in clinical practice	○	○	○	○	○
3. I recognize and appreciate employee efforts toward successful implementation of evidence-based alcohol use interventions	○	○	○	○	○
4. I have removed obstacles to implementing evidence-based alcohol use interventions	○	○	○	○	○
5. I am able to answer clinician's questions about evidence-based alcohol use interventions	○	○	○	○	○
6. I openly address problems regarding the implementation of new processes	○	○	○	○	○

	Not at all	Slight extent	Moderate extent	Great extent	Very great extent
1. The leadership in my practice supports clinicians' efforts to learn about evidence-based alcohol use interventions	○	○	○	○	○
2. The leadership in my practice supports clinicians' efforts to use evidence-based alcohol use interventions in clinical practice	○	○	○	○	○
3. The leadership in my practice recognizes and appreciates employee efforts toward successful implementation of evidence-based alcohol use interventions	○	○	○	○	○
4. The leadership in my practice has removed obstacles to implementing evidence-based alcohol use interventions	○	○	○	○	○

5. My direct supervisor is able to answer my questions about evidence-based alcohol use interventions	○	○	○	○	○
6. My direct supervisor openly addresses problems regarding the implementation of new processes	○	○	○	○	○

Thank you for completing the INSPIRE Implementation Leadership Scale (ILS) survey!

For any stage of implementation, please answer the following questions for what you are currently implementing or will implement.

3. The practice has implemented a process for screening patients for unhealthy alcohol use with a validated question(s).

◯ Not started at this time.
◯ Planning: Implementation in planning or pilot stage
◯ Active: Implementation in process
◯ Full: Implemented across the practice

a. What screening tool(s) is/are used? Please select all that apply.

☐ Single Alcohol Screening Question (SASQ)
☐ AUDIT-C or USAUDIT-C (3 questions)
☐ AUDIT or USAUDIT (10 questions)
☐ Other

If other, please specify. __________________________________

b. Which patients are screened? Please select all that apply.

☐ All patients ages 18 and above
☐ All patients with different age range
☐ Patients with a particular condition
☐ Patients presenting for a health maintenance/preventive care visit
☐ Patients participating in telehealth or phone appointments
☐ Patients participating in web-based/tablet/application-based appointments
☐ Other

Please specify, age range. __________________________________

Please specify, condition. __________________________________

If other, please specify. __________________________________

c. How often are eligible patients screened? Your best estimate is fine.

◯ Never
◯ Up to 25% of time
◯ 26-50% of time
◯ 51-75% of time
◯ 76% of time or more

d. Who is involved in workflow for screening (includes handing out, collecting, entering results in medical record)? Please select all that apply.

- ☐ Self-administered by patient (paper/electronic tool)
- ☐ Clinician (MD, DO, NP, PA)
- ☐ Behavioral health clinician (e.g., psychologist, counselor, social worker, licensed clinical social worker, licensed mental health counselor, chemical dependence and addiction counselor)
- ☐ Other clinical staff providing direct patient care (e.g., RN, LPN/LVN, medical assistant, certified medical assistant, medical technician)
- ☐ Office manager
- ☐ Front/back office staff (those in practice operations and not directly involved in patient care, e.g., receptionists, appointment schedulers, billing staff, data analysts, etc.)
- ☐ Peer provider (e.g., certified peer specialist, peer support specialist, peer recovery coaches)
- ☐ Pharmacist (e.g., PharmD, clinical pharmacist, pharmacy technician)
- ☐ Other

If other, please specify. ______________________

e. What tools/approach(es) do practice members use to document screening? Please select all that apply.

- ☐ No documentation
- ☐ Paper screening tool
- ☐ Paper health record
- ☐ Electronic health record-unstructured notes
- ☐ Electronic health record-a standardized field/screening tool
- ☐ Other

If other, please specify. ______________________

4. The practice has implemented a process for reviewing and interpreting alcohol screening results.

- ◯ Not started at this time.
- ◯ Planning: Implementation in planning or pilot stage
- ◯ Active: Implementation in process
- ◯ Full: Implemented across the practice

a. If so, please describe the process for practice staff/clinicians reviewing and interpreting alcohol screening results. Please select all that apply.

- ☐ EHR/other tool prompts staff/clinician(s) to flag patients for brief intervention
- ☐ Staff/clinician(s) identify patient(s) for clinician(s) to counsel/offer brief intervention
- ☐ Staff/clinician(s) identify patient(s) who needs brief intervention
- ☐ Use results of screen to identify patients for assessment of AUD symptoms
- ☐ Prompt (EHR/other tool) assessment for AUD symptoms
- ☐ Other

If other, please specify. ______________________

5. If a patient screens positive for unhealthy alcohol use, the practice has implemented a process to assess for symptoms of alcohol use disorder.

- ◯ Not started at this time.
- ◯ Planning: Implementation in planning or pilot stage
- ◯ Active: Implementation in process
- ◯ Full: Implemented across the practice

a. If a patient screens positive, what alcohol assessment instrument do they use for further assessment?

◯ DSM-V Criteria Checklist
◯ AUDIT or USAUDIT (10 question)
◯ CAGE
◯ Other

If other, please specify

____

6. The practice has implemented a process for routinely providing feedback to patients on their screening results and brief intervention/counseling to decrease use or risks, reinforce healthy behaviors, or stop drinking alcohol when indicated.

◯ Not started at this time
◯ Planning: Implementation in planning or pilot stage
◯ Active: Implementation in process
◯ Full: Implemented across the practice

7. The practice has implemented a system for initiating/engaging patients with care following identification of unhealthy alcohol use.

◯ Not started at this time
◯ Planning: Implementation in planning or pilot stage
◯ Active: Implementation in process
◯ Full: Implemented across the practice

a. The practice provides the following support to help patients with unhealthy drinking or alcohol use disorder: Please select all that apply.

☐ Brief intervention/brief treatment provided by internal clinician
☐ Brief intervention/brief treatment provided by internal behavioral health provider
☐ Provide patient educational materials
☐ Refer to an external medical or behavioral health provider
☐ Refer to self-help, mutual support, or peer support groups
☐ Refer to external treatment program (inpatient, outpatient, residential)
☐ Prescribe medication for alcohol use disorders
☐ Prescribe medication-assisted therapy
☐ Other services

If other services, please specify.

____

Thank you for completing the SBI/RT and MAT Implementation Checklist!

Change Process Capability Questionnaire

Today's date

____

Practice ID

____

Please indicate the extent to which you agree or disagree that your practice has used the following strategies to improve Unhealthy Alcohol Use care.

	Strongly disagree	Somewhat disagree	Neither agree or disagree	Somewhat agree	Strongly agree
1. Providing information and skills-training	○	○	○	○	○
2. Use of opinion leaders, role modeling, or other vehicles to encourage support for changes	○	○	○	○	○
3. Changing or creating systems in the medical group/clinic that make it easier to provide high quality care	○	○	○	○	○
4. Removal or reduction of barriers to better quality of care	○	○	○	○	○
5. Organizing people into teams focused on accomplishing the change process for improved care	○	○	○	○	○

	Strongly disagree	Somewhat disagree	Neither agree or disagree	Somewhat agree	Strongly agree
6. Delegating to non-physician staff the responsibility to carry out aspects of care that are normally the responsibility of physicians	○	○	○	○	○
7. Providing to those who are charged with implementing improved care the power to authorize and make the desired change	○	○	○	○	○
8. Using periodic measurement of care quality for the purpose of assessing compliance with any new approach to care	○	○	○	○	○
9. Reporting measurements of practice performance for comparison with their peers	○	○	○	○	○
10. Setting goals and benchmarking rates of performance quality at least yearly	○	○	○	○	○

	Strongly disagree	Somewhat disagree	Neither agree or disagree	Somewhat agree	Strongly agree
11. Customizing the implementation of any care changes to each site of care	○	○	○	○	○
12. Use of rapid cycling, piloting, pre-testing, or other vehicles for reducing the risk of negative results from introducing organization-wide change in care	○	○	○	○	○
13. Deliberately designing care improvements so as to make physician participation less work than before	○	○	○	○	○
14. Deliberately designing care improvements to make the care process more beneficial to the patient	○	○	○	○	○

Thank you for completing the INSPIRE Change Process Capability Questionnaire (CPCQ) survey!

SBIRT and MAT Implementation Checklist

Please complete the following survey, which is designed to collect information about your practice for the Unhealthy Alcohol Use initiative. This survey is intended to be administered once at the start of the initiative, at 6 months, and at the end of the study. We strongly encourage you to consult with others in the practice (e.g., physicians, nurses, other staff) to obtain accurate information to complete this survey.

The SBI/RT and MAT Implementation Checklist is an assessment to see what interventions are in place related to alcohol use screening, brief intervention, referral to treatment, and medications for alcohol use disorder. This assessment will help with identifying interventions based on the current workflow.

Today's Date ______________________________

1. Grantee - Midwest

2. Practice ID ______________________________

For any stage of implementation, please answer the following questions for what you are currently implementing or will implement.

3. The practice has implemented a process for screening patients for unhealthy alcohol use with a validated question(s).

◯ Not started at this time.
◯ Planning: Implementation in planning or pilot stage
◯ Active: Implementation in process
◯ Full: Implemented across the practice

a. What screening tool(s) is/are used? Please select all that apply.

☐ Single Alcohol Screening Question (SASQ)
☐ AUDIT-C or USAUDIT-C (3 questions)
☐ AUDIT or USAUDIT (10 questions)
☐ Other

If other, please specify. __________________________________

b. Which patients are screened? Please select all that apply.

☐ All patients ages 18 and above
☐ All patients with different age range
☐ Patients with a particular condition
☐ Patients presenting for a health maintenance/preventive care visit
☐ Patients participating in telehealth or phone appointments
☐ Patients participating in web-based/tablet/application-based appointments
☐ Other

Please specify, age range. __________________________________

Please specify, condition. __________________________________

If other, please specify. __________________________________

c. How often are eligible patients screened? Your best estimate is fine.

◯ Never
◯ Up to 25% of time
◯ 26-50% of time
◯ 51-75% of time
◯ 76% of time or more

d. Who is involved in workflow for screening (includes handing out, collecting, entering results in medical record)? Please select all that apply.

☐ Self-administered by patient (paper/electronic tool)
☐ Clinician (MD, DO, NP, PA)
☐ Behavioral health clinician (e.g., psychologist, counselor, social worker, licensed clinical social worker, licensed mental health counselor, chemical dependence and addiction counselor)
☐ Other clinical staff providing direct patient care (e.g., RN, LPN/LVN, medical assistant, certified medical assistant, medical technician)
☐ Office manager
☐ Front/back office staff (those in practice operations and not directly involved in patient care, e.g., receptionists, appointment schedulers, billing staff, data analysts, etc.)
☐ Peer provider (e.g., certified peer specialist, peer support specialist, peer recovery coaches)
☐ Pharmacist (e.g., PharmD, clinical pharmacist, pharmacy technician)
☐ Other

If other, please specify.

____

e. What tools/approach(es) do practice members use to document screening? Please select all that apply.

☐ No documentation
☐ Paper screening tool
☐ Paper health record
☐ Electronic health record-unstructured notes
☐ Electronic health record-a standardized field/screening tool
☐ Other

If other, please specify.

____

4. The practice has implemented a process for reviewing and interpreting alcohol screening results.

◯ Not started at this time.
◯ Planning: Implementation in planning or pilot stage
◯ Active: Implementation in process
◯ Full: Implemented across the practice

a. If so, please describe the process for practice staff/clinicians reviewing and interpreting alcohol screening results. Please select all that apply.

☐ EHR/other tool prompts staff/clinician(s) to flag patients for brief intervention
☐ Staff/clinician(s) identify patient(s) for clinician(s) to counsel/offer brief intervention
☐ Staff/clinician(s) identify patient(s) who needs brief intervention
☐ Use results of screen to identify patients for assessment of AUD symptoms
☐ Prompt (EHR/other tool) assessment for AUD symptoms
☐ Other

If other, please specify.

____

5. If a patient screens positive for unhealthy alcohol use, the practice has implemented a process to assess for symptoms of alcohol use disorder.

◯ Not started at this time.
◯ Planning: Implementation in planning or pilot stage
◯ Active: Implementation in process
◯ Full: Implemented across the practice

a. If a patient screens positive, what alcohol assessment instrument do they use for further assessment?

◯ DSM-V Criteria Checklist
◯ AUDIT or USAUDIT (10 question)
◯ CAGE
◯ Other

If other, please specify

____

6. The practice has implemented a process for routinely providing feedback to patients on their screening results and brief intervention/counseling to decrease use or risks, reinforce healthy behaviors, or stop drinking alcohol when indicated.

◯ Not started at this time
◯ Planning: Implementation in planning or pilot stage
◯ Active: Implementation in process
◯ Full: Implemented across the practice

7. The practice has implemented a system for initiating/engaging patients with care following identification of unhealthy alcohol use.

◯ Not started at this time
◯ Planning: Implementation in planning or pilot stage
◯ Active: Implementation in process
◯ Full: Implemented across the practice

a. The practice provides the following support to help patients with unhealthy drinking or alcohol use disorder: Please select all that apply.

☐ Brief intervention/brief treatment provided by internal clinician
☐ Brief intervention/brief treatment provided by internal behavioral health provider
☐ Provide patient educational materials
☐ Refer to an external medical or behavioral health provider
☐ Refer to self-help, mutual support, or peer support groups
☐ Refer to external treatment program (inpatient, outpatient, residential)
☐ Prescribe medication for alcohol use disorders
☐ Prescribe medication-assisted therapy
☐ Other services

If other services, please specify.

____

Thank you for completing the SBI/RT and MAT Implementation Checklist!

Implementation Climate Scale (ICS)

Please complete the following 9-item measure, which assesses the degree to which there is a strategic organizational climate supportive of evidence-based practice implementation.

Today's date ______________________

Practice ID ______________________

Please indicate the extent to which you agree with each statement

	Not at all	Slight extent	Moderate extent	Great extent	Very great extent
1. This practice/clinic provides conferences, workshops, or seminars focusing on evidence-based practices	○	○	○	○	○
2. This practice/clinic provides evidence-based practice trainings or in-services	○	○	○	○	○
3. This practice/clinic provides evidence-based practice training materials, journals, etc	○	○	○	○	○

	Not at all	Slight extent	Moderate extent	Great extent	Very great extent
4. Clinicians in this practice/clinic who use evidence-based practices are seen as clinical experts	○	○	○	○	○
5. Clinicians who use evidence-based practices are held in high esteem in this practice/clinic	○	○	○	○	○
6. Clinicians in this practice/clinic who use evidence-based practices are more likely to be promoted	○	○	○	○	○

	Not at all	Slight extent	Moderate extent	Great extent	Very great extent
7. This practice/clinic provides financial incentives for the use of evidence-based practices	○	○	○	○	○

8. The better you are at using evidence-based practices, the more likely you are to get a bonus or a raise	◯	◯	◯	◯	◯
9. This practice/clinic provides the ability to accumulate compensated time for the use of evidence-based practices	◯	◯	◯	◯	◯

Thank you for completing the INSPIRE Implementation Climate Scale (ICS) survey!

Implementation Leadership Scale (ILS)

Please complete the following questions.

Today's date __________________________________

Practice ID __________________________________

Do you serve in a leadership role in this facility (for example, acting as a mentor or in a supervisory role)?

◯ Yes
◯ No

If so, what role? __________________________________

Has the leadership in this facility changed within the last 6 months? ◯ Yes ◯ No

Has the leadership changed within the last 12 months? ◯ Yes ◯ No

The following 6 item survey assesses support in your practice for implementing evidence-based alcohol use interventions. Please complete the 6 items.

Please indicate the extent to which you agree or disagree with each statement.

	Not at all	Slight extent	Moderate extent	Great extent	Very great extent
1. I support clinicians' efforts to learn about evidence-based alcohol use interventions	○	○	○	○	○
2. I support clinicians' efforts to use evidence-based alcohol use interventions in clinical practice	○	○	○	○	○
3. I recognize and appreciate employee efforts toward successful implementation of evidence-based alcohol use interventions	○	○	○	○	○
4. I have removed obstacles to implementing evidence-based alcohol use interventions	○	○	○	○	○
5. I am able to answer clinician's questions about evidence-based alcohol use interventions	○	○	○	○	○
6. I openly address problems regarding the implementation of new processes	○	○	○	○	○

	Not at all	Slight extent	Moderate extent	Great extent	Very great extent
1. The leadership in my practice supports clinicians' efforts to learn about evidence-based alcohol use interventions	○	○	○	○	○
2. The leadership in my practice supports clinicians' efforts to use evidence-based alcohol use interventions in clinical practice	○	○	○	○	○
3. The leadership in my practice recognizes and appreciates employee efforts toward successful implementation of evidence-based alcohol use interventions	○	○	○	○	○
4. The leadership in my practice has removed obstacles to implementing evidence-based alcohol use interventions	○	○	○	○	○

5. My direct supervisor is able to answer my questions about evidence-based alcohol use interventions	○	○	○	○	○
6. My direct supervisor openly addresses problems regarding the implementation of new processes	○	○	○	○	○

Thank you for completing the INSPIRE Implementation Leadership Scale (ILS) survey!
